# Deep sequencing identifies circulating mouse miRNAs that are functionally implicated in manifestations of aging and responsive to calorie restriction

**DOI:** 10.18632/aging.100540

**Published:** 2013-02-28

**Authors:** Joseph M Dhahbi, Stephen R Spindler, Hani Atamna, Amy Yamakawa, Noel Guerrero, Dario Boffelli, Patricia Mote, David IK Martin

**Affiliations:** ^1^ Department of Biochemistry, University of California at Riverside, Riverside, CA 92521, USA; ^2^ Department of Basic Sciences, Neuroscience, The Commonwealth Medical College, Scranton, PA 18510, USA; ^3^ Center for Genetics, Childrens Hospital Oakland Research Institute, Oakland, CA 94609, USA

**Keywords:** Aging, calorie restriction, circulating miRNAs, serum, small RNAs

## Abstract

MicroRNAs (miRNAs) function to modulate gene expression, and through this property they regulate a broad spectrum of cellular processes. They can circulate in blood and thereby mediate cell-to-cell communication. Aging involves changes in many cellular processes that are potentially regulated by miRNAs, and some evidence has implicated circulating miRNAs in the aging process. In order to initiate a comprehensive assessment of the role of circulating miRNAs in aging, we have used deep sequencing to characterize circulating miRNAs in the serum of young mice, old mice, and old mice maintained on calorie restriction (CR). Deep sequencing identifies a set of novel miRNAs, and also accurately measures all known miRNAs present in serum. This analysis demonstrates that the levels of many miRNAs circulating in the mouse are increased with age, and that the increases can be antagonized by CR. The genes targeted by this set of age-modulated miRNAs are predicted to regulate biological processes directly relevant to the manifestations of aging including metabolic changes, and the miRNAs themselves have been linked to diseases associated with old age. This finding implicates circulating miRNAs in the aging process, raising questions about their tissues of origin, their cellular targets, and their functional role in metabolic changes that occur with aging.

## INTRODUCTION

miRNAs are short non-coding RNAs that direct Argonaute protein complexes to mRNAs to trigger degradation or repress translation, thereby inhibiting protein synthesis [1, 2]. A majority of mammalian protein-coding genes are conserved targets of miRNA regulation, and each miRNA has multiple mRNA targets [3]. miRNAs regulate key biological and pathological processes such as differentiation, cell signaling, tumorigenesis, and various disease states [4-11]. As potent post-transcriptional regulators of gene expression and modulators of many important physiological processes, miRNAs are likely participants in the changes associated with aging.

miRNAs can be released into the bloodstream and extracellular spaces. Some secreted miRNAs are packaged in lipid vesicles while others are complexed with high density lipoprotein (HDL) particles or RNA-binding proteins, mainly of the of the Argonaute family [12-15]. The presence of miRNAs as cell-free, nuclease-resistant molecules in the extracellular space raises the question of whether they carry biological functions similar to hormones. There is accumulating evidence that miRNAs take part in cell-to-cell communication to modulate normal and pathologic processes [16-22].

Aging is associated with extensive changes in gene expression reflecting inflammation and stress, and many age-related pathologies specific to tissue types [23-29]. Calorie restriction (CR), a decreased caloric intake without malnutrition, is the only environmental stimulus known to positively interfere with the aging process [30]. CR delays and reverses the gene expression patterns characteristic of age-related dysfunctions [23-29].

Several reports have implicated miRNAs in aging. The lin-4 miRNA regulates lifespan in *Caenorhabditis elegans*, and targets pathways known to govern aging: insulin and insulin-like growth factor-1 signaling and cell cycle checkpoints for DNA damage [31]. Alterations in miRNA levels have been described during mammalian aging and senescence [32-42]. In a mouse model of decreased longevity, miR-1 is thought to target the insulin/insulin-like growth factor-1 pathway by suppressing IGF1, which perhaps reflects a mechanism that functions during normal aging [32, 43]. In mice, humans, and primates, age-induced miRNA changes target tissue-specific functions of aging signaling pathways: oxidative stress defense and mitochondrial maintenance in the liver, apoptosis in the brain, and cell cycle regulation and proliferation in skeletal muscle (reviewed in [32]). Evidence for a relationship between miRNAs and the effects of CR is scant. A single study reported that CR prevented the age-dependent increase of miR-181a-1, miR-30e and miR-34a, along with the reciprocal up-regulation of their target Bcl-2 gene in mouse brain tissues, suggesting that CR decreased apoptosis and induced a gain in neuronal survival [44].

There is some evidence for the involvement of circulating miRNAs in aging. One study reported an increase in miR-34a in plasma, PBMCs, and brains of older mice, with a reciprocal decrease of its target SIRT1, suggesting that miR-34a can be used as biomarker of brain aging [45]. In the second report, an array of 365 miRNAs was used to including centenarians, and in older patients with cardiovascular disease [46]. Another study assessed plasma levels of miRNAs in healthy young and old humans, and reported that transforming growth factor-beta signaling is the main pathway potentially regulated by the differentially abundant circulating miRNAs.

The evidence that miRNAs are present in normal blood, and may be linked to aging, prompted us to carry out a comprehensive assessment of circulating miRNAs in the mouse, in order to observe the effects of age and CR on the levels of these miRNAs. For a study such as this one, deep sequencing has distinct advantages over other available methods: its sensitivity is limited only by the depth of sequencing, it provides accurate counts of each type of miRNA, and it is capable of discovering novel miRNAs. Well-developed informatics tools are able to analyze miRNA sequence datasets, predict the mRNAs regulated by any detected miRNA, and determine the pathways in which targeted mRNAs function. We have used these methods to compare circulating miRNAs in young mice, old mice, and old mice maintained on CR. The results indicate that circulating levels of some miRNAs are markedly increased with age, that CR antagonizes this increase, and that these miRNAs regulate biological processes that are directly relevant to aging.

## RESULTS AND DISCUSSION

### Analysis of circulating miRNA sequencing reads

To investigate the potential effects of aging and calorie restriction (CR) on the circulating levels of miRNAs, we used small RNA-Seq (Illumina reads of 50 nt) to analyze the serum levels of miRNAs from 3 young (7-month) and 3 old (27-month) control mice, and 3 old (27-month) mice subjected to CR from 1 month of age. The raw sequencing reads of circulating miRNAs from the different experimental groups were pre-processed and analyzed with miRDeep2 [47]. After removing the 3' adapter sequences, discarding reads shorter than 18 nucleotides, and aligning the processed reads to the mouse genome, miRDeep2 detects both known and novel miRNAs. The miRDeep2 algorithm is based on the miRNA biogenesis model; it aligns reads to potential hairpin structures in a manner consistent with Dicer processing, and assigns scores that represent the probability that hairpins are true miRNA precursors. In addition to detecting known and novel miRNAs, miRDeep2 estimates their abundance.

### Discovery of novel miRNAs circulating in the mouse serum

**Figure 1:**
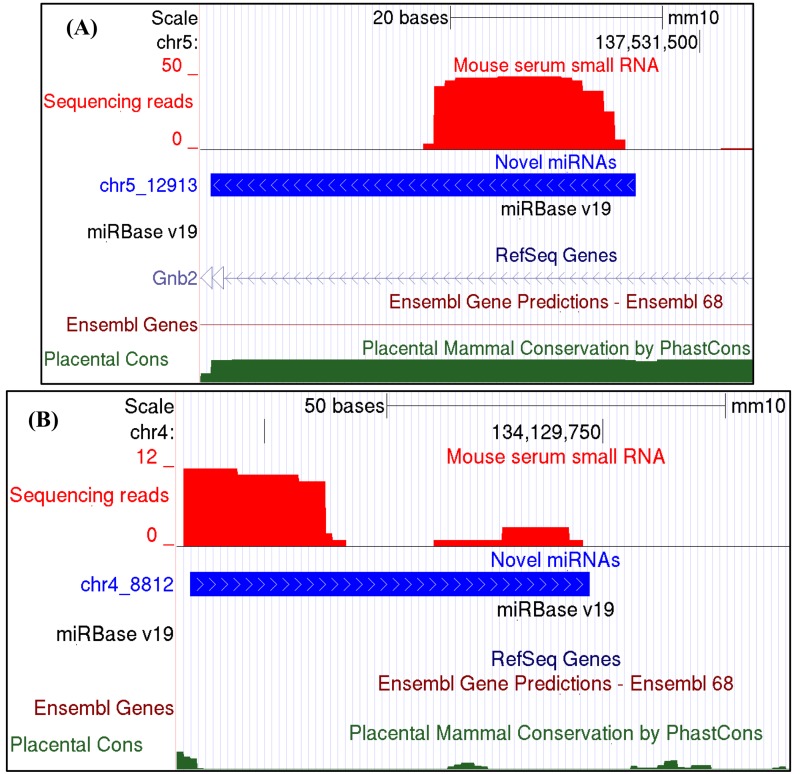
Examples of novel circulating miRNAs discovered with mouse serum small RNA sequencing (**A**) A novel miRNA located in a conserved genomic region and predicted to map to an intron of the *Gnb2* gene as annotated in the RefSeq Genes Track. Shown are screenshots from the UCSC genome browser, displaying the Illumina sequencing reads (red), and the novel precursor miRNA (blue) predicted by miRDeep2 with a provisional id chr5_12913 (see Table S1). (**B**) A novel miRNA with a provisional id chr4_8812 (see Table S1) predicted to map to a genomic region with no known annotated features. UCSC genome browser Ensembl and RefSeq Genes tracks are shown, with no RNAs annotated in the genomic region of this novel miRNA. A miRBase v.19 custom track was uploaded to the UCSC genome browser to show absence of known miRNAs in the genomic regions of the predicted novel miRNAs. The “stacks” of sequence reads identify the mature miRNA. The coverage depth (number of reads, y-axis) shows fewer reads mapping to the star region of the miRNA precursor. The mammalian conservation track is at the bottom (green).

miRDeep2 predicted 79 potential novel miRNAs, and detected 553 known miRNAs, at the relatively stringent score cut-off of 4 and signal-to-noise ratio of 19.6 (Table 1). After filtering of the predicted novel miRNAs by removal of loci matching other RNA genes, and keeping only novel miRNAs with significant randfold p-value, the list was reduced to 23 candidate novel miRNAs (Supplementary Table 1). Illustrative examples of novel miRNAs are depicted in Fig. 1. One of the depicted novel miRNAs is located in a conserved genomic region and is derived from an intron of the *Gnb2* gene. The other example maps to a genomic region with no features annotated in the Ensembl and RefSeq Gene tracks of the UCSC genome browser. The targets (tissues and mRNAs) and functions of these novel miRNAs remain to be discovered.

**Table 1:** Survey of miRDeep2 performance showing the number of novel and known miRNAs and value of signal-to-noise ratio under different score cut-offs ranging from 10 to 1

	Novel miRNAs			Known miRNAs			
miRDeep2 score^1^	Predicted^2^	False positives^3^	True positives^4^	In species^5^	In data^6^	Detected^7^	Signal-to-noise^8^
10	31	5 ± 2	26 ± 2 (85 ± 7%)	1281	848	351 (41%)	27.1
9	31	5 ± 2	26 ± 2 (84 ± 7%)	1281	848	353 (42%)	26.8
8	33	5 ± 2	28 ± 2 (85 ± 7%)	1281	848	359 (42%)	26.7
7	36	5 ± 2	31 ± 2 (85 ± 6%)	1281	848	364 (43%)	26.3
6	43	6 ± 2	37 ± 2 (87 ± 5%)	1281	848	372 (44%)	26.3
5	60	7 ± 3	53 ± 3 (88 ± 4%)	1281	848	515 (61%)	27.9
**4**	**79**	**13 ± 4**	**66 ± 4 (83 ± 5%)**	**1281**	**848**	**553 (65%)**	**19.6**
3	93	39 ± 6	54 ± 6 (58 ± 7%)	1281	848	567 (67%)	8.4
2	139	57 ± 7	82 ± 7 (59 ± 5%)	1281	848	583 (69%)	6.5
1	267	88 ± 10	179 ± 10 (67 ± 4%)	1281	848	645 (76%)	5.5

1 The miRDeep2 score represents the log-odds probability of a sequence being genuine miRNA precursor versus the probability that it is a background hairpin, given the evidence from the data.

2 Number of novel miRNA hairpins with a score ≥ cut-off.

3 Number of false positive miRNA hairpins predicted at this cut-off, as estimated by the miRDeep2 controls. Mean and standard deviation are estimated from 100 rounds of permuted controls.

4 Number of true positive miRNA hairpins is estimated as t = total novel miRNAs - false positive novel miRNAs. The percentage of the predicted novel miRNAs that is estimated to be true positives is calculated as p = t/total novel miRNAs. The number of false positives is estimated from 100 rounds of permuted controls. In each of the 100 rounds, t and p are calculated, generating mean and standard deviation of t and p. The variable p can be used as an estimation of miRDeep2 positive predictive value at the score cut-off.

5 Number of reference mature miRNAs for the human species given as input to miRDeep2.

6 Number of reference mature miRNAs that map perfectly to one or more of precursor candidates that have been excised from the genome by miRDeep2.

7 Number of reference mature miRNAs that map perfectly to one or more of predicted miRNA hairpins that have a score equal to or exceeding the cut-off. The percentage of reference mature miRNAs in data that is detected by miRDeep2 is calculated as s = reference mature miRNAs detected/reference mature miRNAs in data. s can be used as an estimation of miRDeep2 sensitivity at the score cut-off.

8 The signal-to-noise ratio for the given score cut-off is estimated as r = total miRNA hairpins reported / mean estimated false positive miRNA hairpins over 100 rounds of permuted controls.

### Both age and CR alter the abundance of circulating known miRNAs

**Figure 2:**
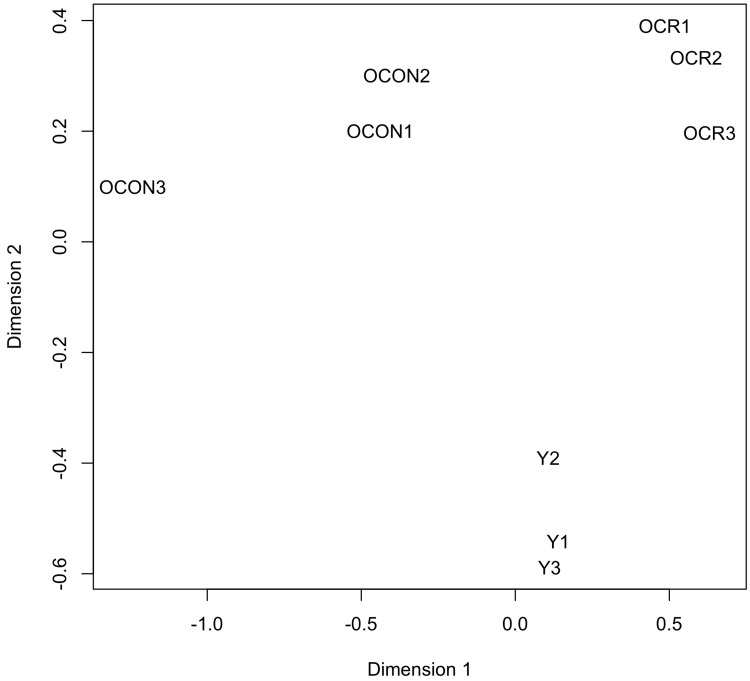
Clustering analysis of the expression values of the circulating miRNAs The plotMDS function of edgeR was used to produce a multi-dimensional scaling plot in which distances reflect the biological coefficient of variation between the miRNA samples. Dimensions 1 and 2 represent the diet and age factors, respectively. The analyzed miRNA samples are from young control (Y1, Y2, and Y3), old control (OCON1, OCON2, and OCON3), and old CR (OCR1, OCR2, and OCR3) mice.

To determine potential effects of age and/or CR on the circulating levels of known miRNAs, the expression values generated by miRDeep2 were analyzed with the Bioconductor package edgeR [48]. The samples were first examined with plotMDS, an edgeR function that produces a multi-dimensional scaling plot in which distances reflect the biological coefficient of variation between samples. One dimension of the plotMDS adequately separated the young group from both old groups (old control and old CR), while the other dimension adequately separated the old control group from the old CR group (Fig. 2). This analysis confirms the homogeneity of the replicates, and reveals distinct effects of age and CR on the abundance of circulating miRNAs.

**Figure 3:**
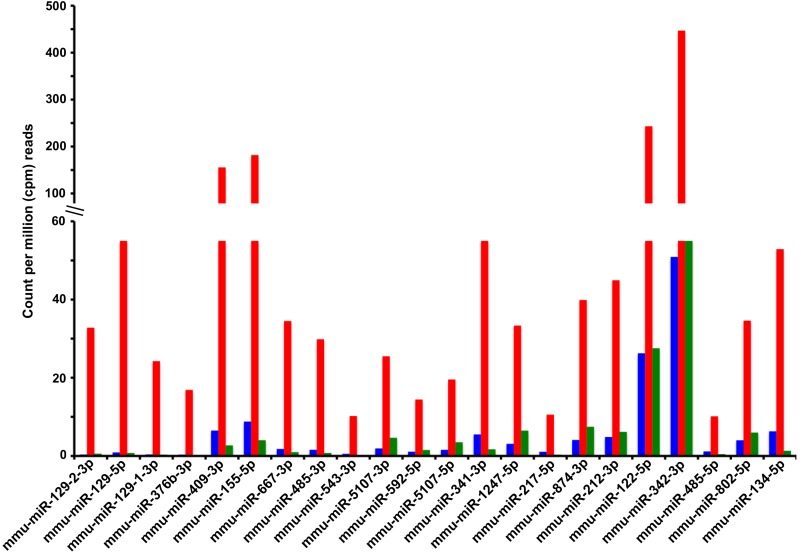
Known miRNAs for which calorie restriction antagonizes an age-associated increase in circulating levels Shown are the serum levels of miRNAs (Y-axis) derived from the miRNA genes indicated in the X-axis (labeled with miRBase v.19 terminology). Serum levels of miRNAs are reported as the average counts per million (cpm) reads in the sequenced libraries from the 3 experimental groups: young control (blue bars), old control (red bars), and old CR (green bars). The fold change and p-values of the age and CR effects on these and other circulating miRNAs are reported in Table 2.

We performed pairwise comparisons between the young and old control groups to measure differences in the circulating miRNAs associated with old age, and pairwise comparisons between the old control and old CR groups, to unravel any potential effect of CR on the age-associated changes in circulating levels of miRNAs. Differences were considered significant if the miRNAs achieved a minimum of 10 counts per million (cpm) reads in at least one of the 3 experimental groups (young, old control, and old CR), the fold change between any two groups was ≥ 1.5, and the p-value of this difference was < 0.05. Application of these criteria revealed that aging increased the circulating levels of 45 known miRNAs, but decreased the circulating levels of only 3 known miRNAs. CR either completely or partially mitigated these age-associated changes. A subset of miRNAs with the largest age-associate fold increases is presented in Fig. 3, while a complete list of all circulating miRNAs affected by both aging and CR are reported in Table 2. Aging was associated with the increase or decrease in the levels of 28 and 44 circulating miRNAs, respectively, without CR having any significant effects on these age-associated changes (Supplementary Table 2). On the other hand, CR altered the circulating levels of a group of miRNAs that were not affected by age; CR decreased the levels of 18, and increased levels of 2 circulating miRNAs (Supplementary Table 3).

**Table 2:** Circulating miRNAs for which the age-associated changes in abundance were prevented by caloric restriction

miRNA	Young (cpm)^1^	Old (cpm)^1^	CR (cpm)^1^	Age FC^2^	Age p-value	CR FC^2^	CR p-value
mmu-miR-376b-3p	0	17	0	46.9	2.3E-05	−141.8	5.5E-07
mmu-miR-543-3p	1	10	0	18.4	1.6E-03	−137.2	2.3E-06
mmu-miR-129-5p	1	68	1	73.8	2.1E-13	−89.3	2.6E-14
mmu-miR-129-1-3p	0	24	0	61.0	2.1E-08	−69.0	6.8E-08
mmu-miR-409-3p	6	156	3	24.0	1.7E-06	−57.6	1.2E-08
mmu-miR-129-2-3p	0	33	1	86.7	2.0E-10	−56.7	1.3E-08
mmu-miR-155-5p	9	182	4	20.7	4.8E-06	−45.2	5.7E-08
mmu-miR-134-5p	6	53	1	8.4	4.0E-03	−39.8	9.9E-07
mmu-miR-485-3p	2	30	1	18.9	1.2E-04	−39.4	1.0E-05
mmu-miR-341-3p	6	61	2	11.1	6.4E-04	−35.7	2.0E-06
mmu-miR-667-3p	2	35	1	19.3	7.1E-06	−35.1	1.9E-07
mmu-miR-217-5p	1	11	0	9.8	9.1E-05	−30.2	8.6E-08
mmu-miR-431-5p	6	41	2	6.9	7.1E-03	−25.4	4.9E-06
mmu-miR-673-5p	4	19	1	5.3	3.2E-02	−22.4	1.4E-05
mmu-miR-485-5p	1	10	0	8.7	7.3E-03	−21.4	4.1E-05
mmu-miR-300-3p	14	91	4	6.7	4.0E-03	−21.2	2.0E-06
mmu-miR-434-3p	82	621	36	7.5	3.4E-04	−17.3	7.4E-07
mmu-miR-668-3p	2	16	1	7.6	2.5E-03	−16.6	1.8E-05
mmu-miR-410-3p	15	76	5	4.9	2.5E-02	−14.3	7.6E-05
mmu-miR-3096a-5p	4	19	2	4.8	6.2E-03	−10.3	3.9E-05
mmu-miR-3096b-5p	4	19	2	4.8	6.3E-03	−10.3	3.9E-05
mmu-miR-592-5p	1	14	2	13.1	6.5E-05	−9.4	1.5E-03
mmu-miR-122-5p	26	243	28	9.3	5.5E-05	−8.8	2.2E-04
mmu-miR-183-5p	17	112	14	6.6	6.4E-04	−8.1	6.4E-04
mmu-miR-212-3p	5	45	6	9.3	4.3E-06	−7.3	6.8E-04
mmu-miR-298-5p	5	21	3	4.0	1.8E-02	−6.5	2.6E-03
mmu-miR-148a-5p	10	40	7	3.8	1.4E-02	−6.1	1.4E-03
mmu-miR-342-3p	51	447	74	8.8	2.1E-09	−6.0	9.4E-05
mmu-miR-802-5p	4	35	6	8.6	6.1E-06	−5.8	2.9E-03
mmu-miR-10a-5p	4936	29790	5239	6.0	5.7E-07	−5.7	8.4E-05
mmu-miR-99b-5p	296	1359	242	4.6	8.3E-04	−5.6	9.6E-04
mmu-miR-182-5p	85	370	68	4.4	3.8E-03	−5.4	4.2E-03
mmu-miR-146a-5p	363	2401	447	6.6	1.9E-06	−5.4	9.3E-04
mmu-miR-10b-5p	4910	20314	3793	4.1	1.1E-03	−5.4	4.0E-04
mmu-miR-192-5p	3908	23200	4628	5.9	8.0E-08	−5.0	2.3E-04
mmu-miR-138-5p	10	58	12	5.7	1.4E-04	−4.9	4.0E-03
mmu-miR-365-3p	12	66	12	5.3	1.1E-03	−5.6	4.8E-03
mmu-miR-6240	10	43	9	4.2	5.7E-03	−5.1	6.5E-03
mmu-miR-5107-3p	2	25	5	13.2	8.4E-08	−5.5	6.5E-03
mmu-miR-5107-5p	2	20	4	12.5	2.3E-06	−5.6	9.2E-03
mmu-miR-5128	16	56	14	3.5	1.7E-02	−4.1	1.2E-02
mmu-miR-1247-5p	3	33	6	10.8	9.6E-07	−5.1	1.3E-02
mmu-miR-874-3p	4	40	7	9.8	7.4E-06	−5.3	1.9E-02
mmu-miR-1943-5p	4	33	7	8.3	3.5E-05	−4.9	2.7E-02
mmu-miR-5115	39	157	43	4.0	5.0E-03	−3.6	3.6E-02
mmu-miR-451a	20664	3725	6222	−5.5	2.9E-13	1.7	5.5E-07
mmu-miR-144-3p	270	92	139	−2.9	5.1E-08	1.5	7.7E-06
mmu-miR-16-2-3p	45	12	18	−3.7	2.2E-08	1.5	3.8E-05

1 Average miRNA read count for the indicated experimental group reported as counts per million (cpm) reads in the sequenced library.

2 Fold change calculated by EdgeR from pairwise comparisons between the young and old control groups for the age effect, or between the old control and old CR groups for the CR effect.

The functional significance of the observed age- and/or CR-associated changes remains to be established: they would be biologically meaningful only if circulating miRNAs are taken up by peripheral tissues, and retain functional mRNA targeting capabilities that regulate gene expression in recipient tissues. Pertinent to this point, circulating miRNAs can bind high-density lipoprotein (HDL), and are taken up by recipient cells where they directly target mRNAs [12]; there are significant differences in the circulating HDL-miRNA profile between normal subjects and patients with familial hypercholesterolemia, and delivery of atherosclerotic HDL-miRNAs to hepatocytes alters the expression of genes related to lipid metabolism, inflammation, and atherosclerosis [12]. In another study, a set of brain miRNAs (miR-137, -181c, -9, -29a/b) was reported to circulate at decreased levels in the serum of Alzheimer's Disease (AD) patients and AD animal models, and these miRNAs are potentially involved in AD through the regulation of ceramides [49]. This evidence for function of circulating miRNAs raises the possibility that they may have utility as diseases markers and even therapeutic targets.

### Pathways relevant to aging are associated with circulating miRNAs whose serum levels are increased with age and decreased by CR

The evidence that circulating miRNAs may be delivered to and directly regulate gene expression in peripheral tissues prompted us to carry out a functional characterization of circulating miRNAs, to assess their potential impact on gene expression in recipient tissues. We focused on the group that shows an age-associated increase in serum levels that is antagonized by CR. First, we identified the cellular mRNAs that may be targeted by miRNAs in the group. Cellular mRNAs were identified with miRDB [50], an algorithm that uses machine learning to predict miRNA targets. Only those mRNAs with a prediction score > 80 were considered, since they are most likely to be real targets [50]. Second, we used DAVID and PANTHER [51, 52] to perform a functional annotation of the potential mRNA targets. The pathways identified by this strategy (Table 3; the pathways in Table 3 are the only pathways with significant scores and p values) reveal that the circulating miRNAs of interest may be involved in the regulation of two GO biological processes (‘positive regulation of macromolecule biosynthetic process’ and ‘negative regulation of apoptosis’), and one PANTHER pathway (‘Wnt signaling’).

**Table 3:** Functional annotation clusters of enriched biological pathways targeted in putative recipient tissues by the circulating miRNAs that were increased by age but decreased by CR

GO biological processes*/PANTHER	Count†	p-value‡	miRNAs***
GO:0010557~positive regulation of macromolecule biosynthetic process	40	1.2E-03	miR-134-5p; miR-148a-5p; miR-192-5p; miR-217-5p; miR-298-5p; miR-365-3p; miR-434-3p
GO:0043066~negative regulation of apoptosis	19	2.0E-02	miR-3096b-5p; miR-376b-3p; miR-431-5p; miR-138-5p
PANTHER: Wnt signaling pathway	26	8.5E-03	miR-592-5p; miR-667-3p; miR-668-3p

* The enrichment score > 1.3 (equivalent to a non-log scale value of 0.05).

† The gene members, which belong to an annotation term.

‡ Fisher Exact p-value representing the degree of enrichment of the GO terms using DAVID or multiple-test P-value obtained by using Bonferroni correction for multiple testing during the PANTHER pathway analysis.

*** The miRNAs predicted to regulate the biological processes in the corresponding functional cluster.

The GO term ‘Positive regulation of macromolecule biosynthetic process’ represents the anabolic pathways that use ATP to synthesize the four classes of macromolecules needed by the cell: polysaccharides, lipids, nucleic acids, and proteins. Both aging and CR are potent modulators of metabolism. Aging decreases macromolecular turnover which may underlie the age-related accumulation of oxidative damage, while CR is thought to extend lifespan by reducing metabolic rate and lowering the production of toxic by-products of metabolism [29]. Aging decreases the expression and activity of enzymes required to mobilize proteins for the production of metabolic energy, while CR increases the catabolism of protein and lipid derived from proteolysis and autophagy to generate substrates for energy generation. Moreover, gene expression profiling studies suggested that CR may retard aging by causing a metabolic shift toward increased protein turnover and decreased macromolecular damage [24-26]. In addition, the most widely accepted theory explaining the lifespan-extending effects of CR is that it shifts energy usage away from growth and reproduction and toward maintenance and stress resistance during times of nutritional stress [53]. Thus the miRNAs whose circulating levels are increased with age, and decreased by CR, are associated with functions that are closely associated with known changes in cellular metabolism that occur with aging. The causal relationship between these miRNAs and manifestations of aging remains to be determined.

The second GO term obtained by the functional analysis of age- and CR-regulated circulating miRNAs is ‘negative regulation of apoptosis’. Aging increases apoptosis in postmitotic tissues, including brain, skeletal and cardiac muscle, and germ cells, impeding the homeostasis of somatic organs and stem cell self-renewal [54-56]. In contrast, the suppression of apoptosis during tumorigenesis may underlie the age-associated increased prevalence of cancers [57]. CR increases apoptosis and decreases cellular proliferation in mitotic tissues, where it selectively eliminates preneoplastic and neoplastic cells, which are more sensitive to apoptosis than normal cells (reviewed in [29]). Three months of CR decrease the number and volume of chemically induced preneoplastic foci by 85%, suggesting that CR may exert its anticarcinogenic activity by preferentially inducing apoptosis in tumors and preneoplastic foci [58, 59]. Given these complex effects of aging and CR on apoptosis, our findings of age- and CR-associated changes in the circulating levels of miRNAs may reveal another layer of apoptosis regulation in potential recipient tissues during aging and in response to CR.

Wnt signaling regulates cell proliferation and differentiation, apoptosis, and stem cell renewal, and plays a complex role in aging (reviewed in [60]). The outcome of its effects on aging depends on whether ß-catenin associates with the transcription factor FOXO to induce senescence, or with TCF/LEF to stimulate stem cell renewal. Since FOXO transcription factors regulate the rate of aging and may mediate the antineoplastic effects of CR [61, 62], it is tempting to speculate that the CR-associated changes in the circulating levels of miRNAs may contribute to fine tuning of Wnt signaling in peripheral tissues, to preclude the age-induced senescence of stem and proliferating cells and delay the onset of age-related disorders.

### Old age diseases are associated with circulating miRNAs whose serum levels are increased with age and decreased by CR

miRNAs regulate a wide range of pathological processes, including age-associated impairments such as neoplasia, inflammation and neurodegeneration [63-66]. There is by now a sizable body of data linking dysregulation of miRNA expression to human disease. Databases [67-69] containing detailed information on microRNA-disease relationships provide a resource for study of the potentially pathogenic role of microRNA dysregulation. We searched the miR2Disease and HMDD databases [67, 68] for associations between specific diseases and the circulating miRNAs whose serum levels we have found to be increased with age and decreased by CR. As shown in Supplementary Table 4, by far the largest number of diseases associated with these circulating miRNAs are cancers; other associated diseases include neurodegenerative, cardiovascular, and inflammatory disorders. All of these pathologies are linked to old age. This association implies that circulating miRNAs whose serum levels are increased with age and decreased by CR may participate in the pathogenesis of age-induced diseases, and that their modulation by CR may underlie the anti-aging effects of CR.

## CONCLUSION

We have carried out a detailed analysis of circulating miRNAs in young mice, old mice, and old mice maintained on calorie restriction (CR). In addition to discovering a set of novel miRNAs, we have comprehensively characterized miRNAs whose serum levels change with age. Serum levels of a large set of miRNAs are increased with age, and CR antagonizes this increase. By identifying cellular mRNAs that have a high probability of being regulatory targets of these miRNAs, and investigating the biological pathways in which the targets function, we have found evidence implicating a set of circulating miRNAs in the aging process and in diseases associated with aging. This intriguing finding raises many questions. One question is the tissues and cells that produce these miRNAs, and by extension the cells on which their presumed regulatory function is exerted; at present nothing is known that would allow us to speculate on this topic. Are the age-regulated miRNAs produced broadly, or by a limited set of cells? Are their targets broad, or specific? Another key question is the causal relationship of these miRNAs to aging processes. Does the increase in circulating levels of these miRNAs actually drive manifestations of aging, or is it merely another effect of aging that is retarded by CR? The GO analysis implicates these miRNAs in metabolic changes that occur with aging, but more investigation will be required to establish if they can cause such changes. This comprehensive analysis of circulating miRNAs, and their relationship to aging and CR, thus presents new opportunities for investigation of the aging process.

## METHODS

### Mice and diets

One-month-old male mice of the long-lived B6C3F1 strain were purchased from Harlan (Indianapolis, IN). One week after arrival, mice were individually housed and randomly assigned to one of two groups, control or calorie restricted (CR). Control mice were fed 93 kcal/wk of a defined control diet (AIN-93M, diet no. F05312, BIO-SERV). CR mice were fed 52.2 kcal/wk of a defined CR diet (AIN-93M, diet no. F05314, BIO-SERV). The CR mice consumed <40% fewer calories than the control group, but the CR diet was enriched so that CR mice consumed approximately the same amount of protein, vitamins, and minerals per gram of body weight as control mice. All mice had free access to water. Mice were maintained at 20-24°C and 50-60% humidity with lights on from 0600 to 1800 h. Sentinel mice were kept in the same room as the experimental mice, and serum samples were screened every 6 months for titers against 11 common pathogens. No positive titers were found during these studies. At 27-months of age, mice were euthanized, and blood was collected through cardiac puncture and processed immediately. A group of control mice were euthanized at 7 months of age and used as a young control group. Each group consisted of 3 mice. The Institutional Animal Care and Use Committee of the University of California, Riverside, approved animal protocols.

### Serum collection, RNA isolation, and small RNA library construction

Immediately after collection, blood was transferred to BD Microtainer tubes (Becton, Dickinson and Company), incubated for 30 min at room temperature to allow blood clotting, and then centrifuged at 5,000 g for 10 min. The serum supernatant was transferred to new tubes, centrifuged at 16,000 g for 15 min to remove any residual cells and cell debris, and stored at −80 °C before use. Isolation of total RNA including small RNA was performed with miRNeasy kit (Qiagen) according to the manufacturer's protocol with the exceptions of mixing 2 mL of Qiazol reagent with 0.4 mL serum, loading the entire aqueous phase onto a single column from the MinElute Cleanup Kit (Qiagen), and eluting the RNA in 20 μL of RNase-free water.

One fourth (5 μL) of the RNA isolated from each serum sample was used to construct sequencing libraries with the Illumina TruSeq Small RNA Sample Prep Kit, following the manufacturer's protocol. Briefly, 3′ and 5′ adapters were sequentially ligated to small RNA molecules and the obtained ligation products were subjected to a reverse transcription reaction to create single stranded cDNA. To selectively enrich those fragments that have adapter molecules on both ends, the cDNA was amplified with 15 PCR cycles using a common primer and a primer containing an index tag; this allows multiplexing and sequencing of different samples in a single lane of a flowcell. The amplified cDNA constructs were gel purified, and validated by checking the size, purity, and concentration of the amplicons on the Agilent Bioanalyzer High Sensitivity DNA chip. The libraries were pooled in equimolar amounts, and sequenced on an Illumina HiSeq 2000 instrument to generate 50 base reads. Image deconvolution and quality values calculation were performed using the modules of the Illumina pipeline.

### MiRDeep2 analysis of sequencing reads

Raw sequencing reads were analyzed with miRDeep2 [47]. Briefly, miRDeep2 pre-processed raw sequencing reads by removing the 3' adapter sequence and discarding reads shorter than 18 nucleotides, before aligning reads to the mouse genome (NCBI37/mm10). For the purpose of analyzing the sequenced miRNAs, the known miRNA input was from miRBase v.19, and Rattus norvegicus was designated as the related species. MiRDeep2 estimates expression levels of known miRNAs, and identifies novel miRNAs. Small RNAs identified by short read sequencing are derived from longer RNAs, and may or may not be true miRNAs. The miRDeep2 algorithm is a probabilistic algorithm based on the miRNA biogenesis model, and is designed to detect miRNAs from deep sequencing reads. It aligns reads to potential hairpin structures in a manner consistent with Dicer processing of hairpins to produce mature miRNAs, and assigns scores that measure the probability that hairpins are true miRNA precursors. A small RNA derived from the 5' end of a predicted precursor is considered to be a miRNA if it is highly abundant relative to small RNAs derived from loop and star regions of the precursor, and less likely to be a true miRNA if it is present in similar proportions to the loop and star. The miRDeep2 algorithm uses this principle to produce a log-odds score that a small RNA is a true miRNA; it outputs a scored list of known and novel miRNAs as well as their expression levels.

### Statistical analysis of differential miRNA expression

In addition to identifying mature miRNAs in deep sequenced small RNA samples, miRDeep2 also generates expression values for the detected miRNAs. To test for differential abundance of circulating miRNAs between the experimental groups (young control, old control, and old CR), expression data for known miRNAs produced by miRDeep2 were used as input for the Bioconductor package edgeR [48] to quantify the differential abundance of circulating miRNAs. The algorithm of edgeR fits a negative binomial model to the count data, estimates dispersion, and measures differences using the generalized linear model likelihood ratio test which is recommended for experiments with multiple factors, such as the simultaneous analysis of the effects of age and diet in our study. The fitted count data was analyzed by performing pairwise comparisons between the different experimental groups. Young and old control groups were compared to measure the differential abundance in circulating miRNAs associated with old age. Old control and old CR groups were compared to determine whether CR has an effect on any age-associated changes. The differentially abundant miRNAs identified by edgeR were further filtered to remove miRNAs with less than 10 counts per million (cpm) in at least one of the 3 experimental groups.

### Prediction and functional annotation of peripheral genes potentially targeted by differentially abundant circulating miRNAs

mRNA transcripts that are potentially targeted by circulating miRNAs were extracted from miRDB database, which uses machine learning to predict miRNA targets; we included only mRNAs with prediction score > 80, since they are most likely to be real [50]. The predicted mRNAs were functionally annotated with DAVID and PANTHER [51, 52]. PANTHER uses pathways compiled by experts to find pathways that are overrepresented in a list of genes by comparing it to a reference list, e.g. all genes in the mouse genome as used here. A Bonferroni correction for multiple testing was applied to the analysis. We also used DAVID to interrogate representation in Gene Ontology (GO). The DAVID algorithm measures the similarities among GO terms based on the extent of their associated genes and assembles the similar and redundant GO terms into annotation clusters. Each GO term in a cluster is assigned a Fisher Exact p-value representing the degree of enrichment of the GO term in the input gene list. Each cluster is assigned an enrichment score to rank its biological significance. Thus, a biologically significant cluster (high enrichment score) is generated only when most of its GO term members have significant enrichment values (low Fisher Exact p-values). The resulting clusters were further curated to keep only GO terms with p-values < 0.05.

## SUPPLEMENTAL DATA








