# Supplementary material for: Deep sequencing identifies circulating mouse miRNAs that are functionally implicated in manifestations of aging and responsive to calorie restriction
Source: Aging (Albany NY). 2013 Feb 28;5(2):130–41. doi: 10.18632/aging.100540 (PMC3616200; doi:10.18632/aging.100540)
Supplement: Supplementary file 2 [file aging-05-130-s002.docx]

**Supplementary Table 2**. miRNAs displaying an age-associated change in serum abundance that is NOT antagonized by caloric restriction^1^.

| **miRNA** | **Young (cpm)^2^** | **Old (cpm)^2^** | **CR (cpm)^2^** | **Age FC^3^** | **Age p-value** | **CR FC^3^** | **CR p-value** |
| --- | --- | --- | --- | --- | --- | --- | --- |
| mmu-miR-1843b-3p | 8 | 39 | 27 | 4.6 | 5.4E-03 | -1.4 | 1.4E-01 |
| mmu-miR-375-3p | 410 | 2484 | 1648 | 6.1 | 7.6E-06 | -1.5 | 1.3E-01 |
| mmu-miR-330-3p | 10 | 34 | 23 | 3.5 | 3.4E-02 | -1.5 | 2.9E-01 |
| mmu-miR-423-5p | 1758 | 8095 | 5012 | 4.6 | 8.9E-05 | -1.6 | 8.9E-02 |
| mmu-miR-151-3p | 1540 | 4646 | 2346 | 3.0 | 1.8E-02 | -2.0 | 7.0E-01 |
| mmu-miR-744-5p | 308 | 1069 | 512 | 3.5 | 8.4E-03 | -2.1 | 6.8E-01 |
| mmu-miR-1964-3p | 28 | 86 | 40 | 3.1 | 3.8E-02 | -2.2 | 9.9E-01 |
| mmu-miR-664-5p | 7 | 38 | 15 | 5.8 | 1.9E-04 | -2.5 | 5.8E-01 |
| mmu-miR-511-5p | 10 | 44 | 17 | 4.3 | 9.5E-03 | -2.5 | 8.4E-01 |
| mmu-miR-1249-3p | 21 | 185 | 73 | 8.7 | 1.3E-05 | -2.5 | 8.8E-01 |
| mmu-miR-361-5p | 55 | 161 | 63 | 2.9 | 4.3E-02 | -2.5 | 5.7E-01 |
| mmu-miR-3473d | 6 | 25 | 10 | 4.2 | 1.3E-02 | -2.6 | 7.8E-01 |
| mmu-miR-423-3p | 590 | 3366 | 1226 | 5.7 | 6.7E-06 | -2.7 | 3.7E-01 |
| mmu-miR-425-3p | 6 | 26 | 9 | 4.3 | 1.1E-02 | -2.9 | 3.0E-01 |
| mmu-miR-1981-5p | 14 | 74 | 25 | 5.1 | 9.8E-04 | -3.0 | 5.1E-01 |
| mmu-miR-877-5p | 10 | 43 | 14 | 4.4 | 2.0E-03 | -3.0 | 3.0E-01 |
| mmu-miR-877-3p | 4 | 22 | 7 | 5.7 | 1.7E-03 | -3.0 | 3.6E-01 |
| mmu-miR-671-3p | 18 | 84 | 27 | 4.7 | 4.5E-04 | -3.1 | 2.2E-01 |
| mmu-miR-5119 | 2 | 18 | 6 | 11.9 | 1.4E-05 | -3.1 | 2.5E-01 |
| mmu-miR-6243 | 20 | 74 | 23 | 3.8 | 7.6E-03 | -3.2 | 1.2E-01 |
| mmu-miR-150-5p | 2167 | 10809 | 3360 | 5.0 | 9.6E-06 | -3.2 | 1.1E-01 |
| mmu-miR-99b-3p | 7 | 31 | 10 | 4.4 | 1.1E-02 | -3.2 | 1.9E-01 |
| mmu-miR-92b-3p | 64 | 288 | 83 | 4.5 | 9.2E-04 | -3.5 | 9.4E-02 |
| mmu-miR-28a-3p | 20 | 68 | 19 | 3.4 | 2.6E-02 | -3.6 | 6.4E-02 |
| mmu-miR-187-3p | 6 | 34 | 9 | 5.8 | 6.7E-04 | -3.7 | 1.6E-01 |
| mmu-miR-615-3p | 4 | 15 | 4 | 3.7 | 4.3E-02 | -3.7 | 7.3E-02 |
| mmu-miR-5124a | 3 | 24 | 6 | 8.5 | 2.2E-04 | -3.9 | 6.3E-02 |
| mmu-miR-150-3p | 6 | 69 | 17 | 12.1 | 3.7E-08 | -4.1 | 6.0E-02 |
| mmu-miR-106b-3p | 791 | 355 | 509 | -2.2 | 3.3E-06 | 1.4 | 8.7E-06 |
| mmu-miR-15a-3p | 18 | 7 | 10 | -2.6 | 9.7E-05 | 1.3 | 1.6E-03 |
| mmu-miR-98-5p | 131 | 18 | 24 | -7.2 | 1.4E-11 | 1.3 | 9.3E-04 |
| mmu-miR-15b-3p | 103 | 50 | 65 | -2.1 | 4.2E-05 | 1.3 | 1.7E-04 |
| mmu-miR-144-5p | 64 | 26 | 33 | -2.5 | 1.3E-06 | 1.3 | 1.9E-04 |
| mmu-miR-181c-5p | 774 | 390 | 493 | -2.0 | 2.4E-06 | 1.3 | 7.9E-05 |
| mmu-miR-106b-5p | 473 | 164 | 206 | -2.9 | 1.3E-08 | 1.3 | 4.5E-05 |
| mmu-let-7i-5p | 3582 | 1479 | 1775 | -2.4 | 3.5E-08 | 1.2 | 4.5E-05 |
| mmu-let-7j | 897 | 429 | 512 | -2.1 | 1.3E-06 | 1.2 | 8.9E-05 |
| mmu-miR-93-5p | 3750 | 1750 | 2040 | -2.1 | 1.5E-06 | 1.2 | 7.0E-05 |
| mmu-let-7d-5p | 333 | 169 | 196 | -2.0 | 5.4E-06 | 1.2 | 9.0E-05 |
| mmu-miR-186-5p | 2053 | 912 | 1042 | -2.3 | 3.8E-07 | 1.1 | 1.2E-04 |
| mmu-miR-16-5p | 31684 | 12292 | 13872 | -2.6 | 1.1E-07 | 1.1 | 1.5E-04 |
| mmu-miR-499-5p | 32 | 2 | 2 | -15.5 | 3.6E-15 | 1.1 | 1.9E-02 |
| mmu-miR-15b-5p | 135 | 82 | 91 | -1.6 | 5.7E-05 | 1.1 | 2.2E-04 |
| mmu-miR-17-5p | 100 | 61 | 62 | -1.6 | 1.1E-04 | 1.0 | 1.0E-03 |
| mmu-miR-18a-5p | 20 | 14 | 14 | -1.5 | 3.5E-03 | 1.0 | 6.4E-03 |
| mmu-miR-421-3p | 354 | 202 | 202 | -1.8 | 2.5E-04 | -1.0 | 2.7E-03 |
| mmu-miR-15a-5p | 1148 | 609 | 603 | -1.9 | 1.8E-05 | -1.0 | 1.1E-03 |
| mmu-miR-20a-5p | 38 | 21 | 21 | -1.8 | 5.1E-05 | -1.0 | 2.6E-03 |
| mmu-miR-350-3p | 14 | 7 | 7 | -2.0 | 4.0E-04 | -1.0 | 1.8E-02 |
| mmu-miR-30e-5p | 8888 | 6012 | 5853 | -1.5 | 2.3E-05 | -1.0 | 2.3E-04 |
| mmu-let-7g-5p | 1510 | 581 | 520 | -2.6 | 2.2E-08 | -1.1 | 2.8E-03 |
| mmu-miR-30c-5p | 1616 | 851 | 758 | -1.9 | 1.6E-06 | -1.1 | 1.6E-03 |
| mmu-let-7c-5p | 2026 | 1265 | 1081 | -1.6 | 9.8E-05 | -1.2 | 5.6E-03 |
| mmu-miR-133b-3p | 131 | 18 | 15 | -7.4 | 3.2E-17 | -1.2 | 1.3E-02 |
| mmu-miR-378a-5p | 45 | 13 | 11 | -3.5 | 6.6E-08 | -1.2 | 3.7E-02 |
| mmu-miR-374b-5p | 19 | 6 | 5 | -3.1 | 2.8E-06 | -1.2 | 6.0E-02 |
| mmu-miR-133a-3p | 6211 | 1715 | 1331 | -3.6 | 1.7E-12 | -1.3 | 9.5E-03 |
| mmu-miR-142-3p | 127 | 79 | 60 | -1.6 | 1.7E-04 | -1.3 | 3.7E-02 |
| mmu-miR-26b-5p | 271 | 106 | 76 | -2.5 | 9.0E-08 | -1.4 | 5.2E-02 |
| mmu-miR-3969 | 114 | 44 | 6 | -2.6 | 2.5E-03 | -7.8 | 2.8E-03 |
| mmu-miR-3964 | 19 | 8 | 1 | -2.4 | 1.4E-03 | -6.2 | 4.2E-03 |
| mmu-miR-1a-3p | 11 | 1 | 0 | -8.5 | 2.5E-06 | -6.3 | 1.1E-01 |
| mmu-miR-340-5p | 83 | 55 | 35 | -1.5 | 9.9E-05 | -1.6 | 2.0E-01 |
| mmu-miR-378d | 677 | 254 | 82 | -2.7 | 3.1E-06 | -3.1 | 2.5E-01 |
| mmu-miR-195a-5p | 27 | 17 | 5 | -1.6 | 5.2E-04 | -3.3 | 2.6E-01 |
| mmu-miR-296-5p | 12 | 8 | 5 | -1.5 | 1.7E-02 | -1.7 | 2.8E-01 |
| mmu-miR-34c-5p | 26 | 10 | 2 | -2.6 | 5.2E-03 | -4.6 | 2.8E-01 |
| mmu-miR-30e-3p | 50 | 26 | 13 | -1.9 | 7.5E-06 | -2.0 | 6.2E-01 |
| mmu-miR-490-3p | 20 | 9 | 5 | -2.1 | 3.2E-04 | -2.0 | 7.3E-01 |
| mmu-miR-133a-5p | 10 | 3 | 1 | -3.6 | 8.7E-07 | -2.3 | 8.8E-01 |
| mmu-miR-208a-3p | 21 | 3 | 2 | -6.3 | 6.1E-10 | -2.0 | 8.8E-01 |
| mmu-miR-378b | 13 | 8 | 3 | -1.7 | 8.9E-04 | -2.6 | 1.0E+00 |

^1^Changes were considered significant if the fold change ≥ 1.5 and the p-value < 0.05.

^2^Average miRNA read count for the indicated experimental group reported as counts per million (cpm) reads in the sequenced library.

^3^Fold change calculated by EdgeR from pairwise comparisons between the young and old control groups for the age effect, or between the old control and old CR groups for the CR effect.
